# TGFβR2 is a major target of miR-93 in nasopharyngeal carcinoma aggressiveness

**DOI:** 10.1186/1476-4598-13-51

**Published:** 2014-03-08

**Authors:** Xiaoming Lyu, Weiyi Fang, Longmei Cai, Hang Zheng, Yanfen Ye, Lan Zhang, Jinbang li, Hong Peng, William C S Cho, Ena Wang, Francesco M Marincola, Kaitai Yao, Hongbing Cai, Jiliang Li, Xin Li

**Affiliations:** 1 Cancer Research Institute and the Provincial Key Laboratory of Functional Proteomics, Southern Medical University, Guangzhou, China; 2 School of Chinese Traditional Medicine, Southern Medical University, Guangzhou, China; 3 Department of Otorhinolaryngology, Nanfang Hospital, Southern Medical University, Guangzhou, China; 4 Departments of Oncology, Nanfang Hospital, Southern Medical University, Guangzhou, China; 5 Department of Clinical Oncology, Queen Elizabeth Hospital, Guangzhou, Hong Kong; 6 Infectious Disease and Immunogenetics Section, Department of Transfusion Medicine, Clinical Center, National Institutes of Health, Bethesda, USA; 7 School of Biotechnology, Southern Medical University, Guangzhou, China; 8 Molecular Oncology Laboratories, Department of Oncology, Weatherall Institute of Molecular Medicine, University of Oxford, John Radcliffe Hospital, Oxford, UK

**Keywords:** miR-93, TGFβR2, Aggressiveness, PI3K/Akt, Nasopharyngeal carcinoma

## Abstract

**Background:**

MiR-17-92 cluster and its paralogues have emerged as crucial regulators of many oncogenes and tumor suppressors. Transforming growth factor-β receptor II (TGFβR2), as an important tumor suppressor, is involved in various cancer types. However, it is in cancer that only two miRNAs of this cluster and its paralogues have been reported so far to regulate TGFβR2. MiR-93 is oncogenic, but its targetome in cancer has not been fully defined. The role of miR-93 in nasopharyngeal carcinoma (NPC) still remains largely unknown.

**Methods:**

We firstly evaluated the clinical signature of TGFβR2 down-regulation in clinical samples, and next used a miRNA expression profiling analysis followed by multi-validations, including Luciferase reporter assay, to identify miRNAs targeting TGFβR2 in NPC. *In vitro* and *in vivo* studies were performed to further investigate the effects of miRNA-mediated TGFβR2 down-regulation on NPC aggressiveness. Finally, mechanism studies were conducted to explore the associated pathway and genes influenced by this miRNA-mediated TGFβR2 down-regulation.

**Results:**

TGFβR2 was down-regulated in more than 50% of NPC patients. It is an unfavorable prognosis factor contributing to clinical NPC aggressiveness. A cluster set of 4 TGFβR2-associated miRNAs was identified; they are all from miR-17-92 cluster and its paralogues, of which miR-93 was one of the most significant miRNAs, directly targeting TGFβR2, promoting cell proliferation, invasion and metastasis *in vitro* and *in vivo*. Moreover, miR-93 resulted in the attenuation of Smad-dependent TGF-β signaling and the activation of PI3K/Akt pathway by suppressing TGFβR2, further promoting NPC cell uncontrolled growth, invasion, metastasis and EMT-like process. Impressively, the knockdown of TGFβR2 by siRNA displayed a consentaneous phenocopy with the effect of miR-93 in NPC cells, supporting TGFβR2 is a major target of miR-93. Our findings were also substantiated by investigation of the clinical signatures of miR-93 and TGFβR2 in NPC.

**Conclusion:**

The present study reports an involvement of miR-93-mediated TGFβR2 down-regulation in NPC aggressiveness, thus giving extended insights into molecular mechanisms underlying cancer aggressiveness. Approaches aimed at blocking miR-93 may serve as a promising therapeutic strategy for treating NPC patients.

## Introduction

Transforming growth factor-β (TGF-β) signaling has tumor suppressive and pro-oncogenic functions in accordance with tumor stage [1]. Its abrogation is always accomplished by either blockade of TGF-β responses or the acquisition of genetic alterations and epigenetic modifications in its components including transforming growth factor-β receptor II (TGFβR2) [2]. TGFβR2, as a tumor-suppressor gene [3,4] is downregulated in multiple cancer types including head and neck squamous cell carcinoma (HNSCC) and is generally related to cancer aggressive behavior [5-9]. Cancer cells always lose their sensitivity to TGF-β-mediated growth inhibitory responses upon TGFβR2 down-regulation [10]. The mechanisms underlying the downregulation of TGFβR2 expression in cancer cells have been investigated, showing that repressed expression of TGFβR2 in microsatellite instability-high colorectal cancer and esophageal adenocarcinoma involves hypermethylation of the TGFβR2 promoter region [11,12]. However, TGFβR2 promoter methylation is not frequent in some cancers such as Head and neck squamous cell carcinoma (HNSCC) (11.4%) [13], although there is a frequent loss of TGFβR2, suggesting that other mechanisms may contribute to the downregulation of TGFβR2 expression.

MiRNAs have emerged as important regulators of gene expression. They can modulate multiple biological processes by inducing translational inhibition and/or mRNA degradation of protein-coding genes. The miR-17-92 cluster is among the best-studied miRNA clusters in carcinogenesis, also known as ‘oncomiR-1’ [14]. It has pivotal roles in a variety of cancers such as colorectal cancer [15-17], breast cancer [18-21], pancreatic cancer [22,23], ovarian cancer [24], lung cancer [25,26], and hepatocellular carcinoma [27-29]. MiR-93, derived from a paralogue (miR-106b-25) of miR-17-92 cluster, is up-regulated in various types of cancers [30-32]. The identified targets of miR-93 include LATS2 [33], AICDA [34], ITGB8 [35], PTEN [36], VEGFA [37], TP53INP1 [38], DAB2 [39], etc., suggesting that miR-93 may play oncogenic roles through diverse mechanisms. However, the targetome of miR-93 in cancer has not been fully defined so far. The role of miR-93 in nasopharyngeal carcinoma (NPC) still remains largely unknown.

We previously found a reduced TGFβR2 expression in NPC [40], which was subsequently supported by the findings from Zhang et al. [41]. Although many miRNAs (For example miR-26a [42,43], miRNA-18a [44], miR-18b [45], miR-218 [46], miR-216b [47], miR-663 [48], miR-155 [49], miR-205 [50], EBV-encoded miRNAs [51], etc.) have been reported to be involved in NPC carcinogenesis, no evidence was given for their associations with TGFβR2 down-regulation.

In the current study, using a miRNA expression profiling analysis in NPC samples stratified by TGFβR2 expression level, we identified a cluster set of 4 TGFβR2-associated miRNAs (miR-93, miR-20a, miR-20b, and miR-18a). They are all from miR-17-92 cluster and its paralogues, of which miR-93 was one of the most significant miRNAs. We demonstrated that miR-93 could directly suppress TGFβR2 and facilitate NPC aggressiveness (NPC cell growth, metastasis and EMT-like process). Mechanistic investigation disclosed that miR-93 could result in attenuated Smad-dependent TGF-B pathway and activated PI3K/Akt pathway by suppressing TGFβR2.

Thus, our study first reports a miR-93-mediated TGFβR2 down-regulation in NPC, extending novel mechanistic insights into the role of miR-93 in cancer aggressiveness. Blocking of miR-93 may be a promise for cancer therapy.

## Results

### TGFβR2 down-regulation is associated with NPC aggressiveness

**Figure 1:**
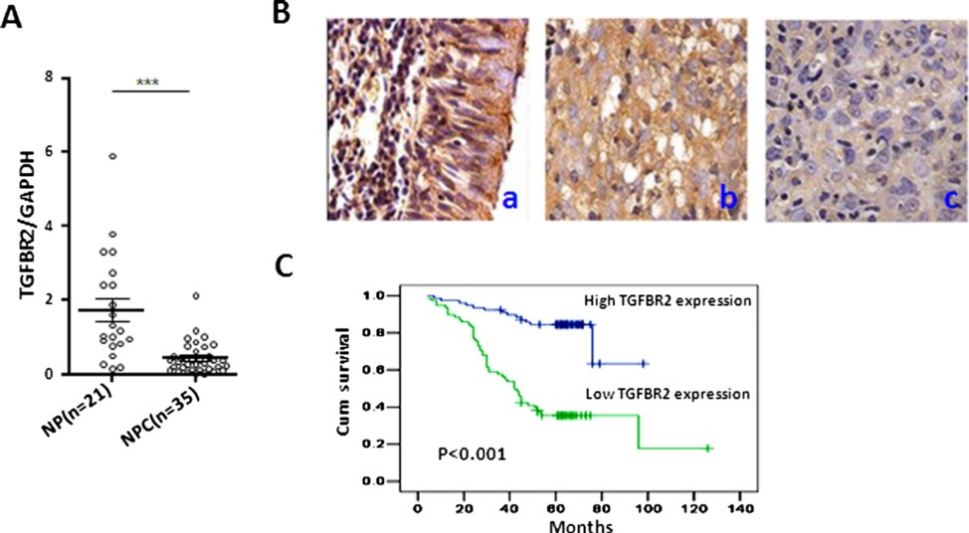
**TGFβR2 down-regulation is associated with NPC aggressiveness. (A)** TGFβR2 mRNA expression was detected by qRT-PCR in 21 NP tissues and 35 NPC tissues. Values represent mean ± SD, ***P < 0.001. **(B)** Representative TGFβR2 IHC images (400×). a. Normal nasopharynx epithelium, b. NPC with high TGFβR2 expression, c. NPC with low TGFβR2 expression. **(C)** Kaplan-Meier survival analysis in NPC patients according to TGFβR2 protein expression levels. The log-rank test was used to calculate p values (p < 0.001).

Our previous study reported a down-regulated TGFβR2 expression in NPC [40], so we initially confirmed it in the present study. TGFβR2 expression was indeed observed to be significantly reduced in NPC patients relative to non-cancerous nasopharyngeal (NP) (Figure 1A). Next, we investigated the clinical signature of TGFβR2 down- regulation using IHC in additional 300 clinical NPC samples. TGFβR2 protein was down-regulated in 51.9% (108/208) of NPC, 38.5% (5/13) of atypical hyperplasia, 9.1% (2/22) of normal squamous epithelium, and 5.3% (3/57) of normal epithelium, displaying a gradual reduction trend from normal epithelium to NPC (Table 1). Pathological analysis showed that the expression level of TGFβR2 is negatively correlated with T classification (the size of the primary tumor and whether it has invaded nearby tissue), N classification (the degree of spread to regional lymph nodes), and clinical stage of NPC patients (Additional file 1: Table S1). Kaplan-Meier survival analysis revealed that TGFβR2 expression was significantly correlated with patient overall survival (Figure 1B, C and Additional file 2: Figure S1). Multivariate survival analysis using the Cox's proportional hazards model showed a close correlation of low TGFβR2 protein expression with clinical prognosis (Additional file 1: Table S2).

**Table 1:** A gradual reduction trend of TGFβR2 protein expression from normal epithelium to NPC

**Group**	**Protein expression (n)**			**P value**					
	**Total**	**Low**	**High**	**S/N/A/C**	**S/N**	**A/N**	**C/N**	**A/C**	**S/C**
S	22	2	20	<0.001*	0.614^#^	0.005^#^	<0.001^#^	0.401^#^	<0.001^#^
N	57	3	54						
A	13	5	8						
C	208	108	100						

S: Squamous epithelium; N: Normal epithelium; A: Atypical hyperplasia; C: NPC.

n: Number of cases. *Kruskal Wallis Test; ^#^Chi-square Test.

Subsequently, we examined the TGFβR2 expressions in 4 NPC cell lines (CNE1, CNE2, 5-8 F, 6-10B), pooled NPC tissues, and an immortalized primary nasopharyngeal epithelial cell line (NP69). The mRNA and protein expressions of TGFβR2 were generally down-expressed in NPC cells and NPC tissues relative to NP69. The more metastatic or aggressive NPC cells (such as CNE-2 and 5-8 F) had relatively lower TGFβR2 expression than that of NPC cells (such as CNE1 and 6-10B) with less metastatic or aggressive potential (Additional file 2: Figure S2).

Collectively, these data support a close relevance of TGFβR2 down-regulation to NPC aggressiveness.

### MiR-93 suppresses TGFβR2 in NPC

**Figure 2:**
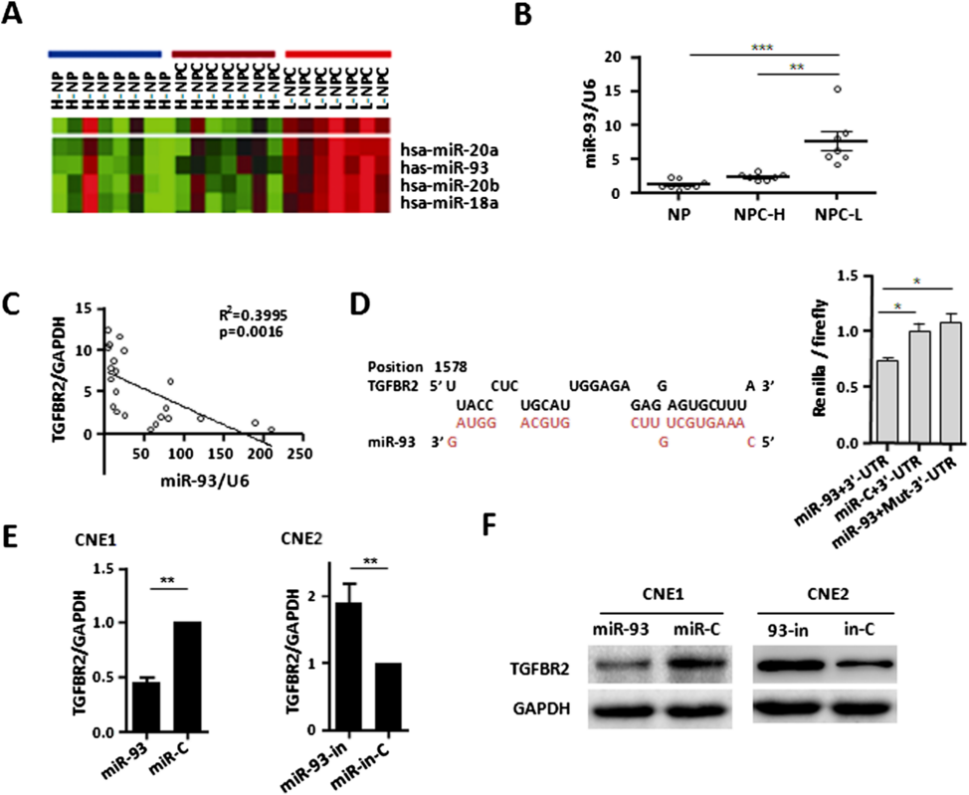
**miR-93 directly suppresses TGFβR2 in NPC. (A)** miRNA expression profiling analysis in three subgroups of clinical samples including 8 NP with high TGFβR2 expression (H-NP, a blue bar on top), 7 NPC with high TGFβR2 expression (H-NPC, a brown bar on top) and 7 NPC with low TGFβR2 expression (L-NPC, a red bar on top). **(B)** qRT-PCR validation of miR-93 expression in 22 clinical tissue samples. Values represent mean ± SD, n = 3. **P < 0.01. ***P < 0.001. **(C)** Correlation analysis between TGFβR2 and miR-93 in 22 clinical tissue samples. **(D)** RNAhybrid prediction of the binding sites of miR-93 in the 3′ UTR of TGFβR2. Luciferase activities of dual luciferase reporter carrying a wild-type or mutant of TGFβR2 3′ UTR in the indicated cells in triplicate. Values represent mean ± SD. *P < 0.05. **(E, F)** The detection of TGFβR2 mRNA and protein expressions by qRT-PCR and Western blot in CNE1 and CNE2 cells transfected with miR-93 mimic and inhibitor respectively. Values represent mean ± SD, n = 3. **P < 0.01.

To investigate if miRNAs are involved in regulation of TGFβR2 expression in NPC, we first selected 22 clinical samples and stratified them into 3 groups based on the mRNA level of TGFβR2: (1) high expression NP group (H-NP) containing 8 NP samples, (2) high expression NPC group (H-NPC) containing 7 NPC samples, and (3) low expression NPC group (L-NPC) containing 7 NPC samples (Additional file 2: Figure S3). We then conducted a miRNA expression profiling analysis for these 3 groups. As shown in Figure 2A and Additional file 1: Table S3, significantly higher expressions of miR-93, miR-20a, miR-20b, and miR-18a were observed in the L-NPC group. They are clustered together and all from miR-17-92 cluster and its paralogues. Of them, miR-93 gained our attention because it shows an oncogenic potential but it has been unclear whether miR-93 could regulate TGFBR2 in cancers, and no studies reported its roles and target genes in NPC. We applied qRT-PCR to confirm that miR-93 was highly expressed in NPC samples (Figure 2B) and 5 NPC cell lines (Additional file 2: Figure S4), and also observe that TGFβR2 expression was inversely correlated with miR-93 expression (R^2^ = 0.3995) (Figure 2C, Additional file 2: Figure S3), suggesting that miR-93 may regulate TGFβR2.

To determine whether TGFβR2 was a direct target of miR-93, we performed a bioinformatic analysis using RNAhybrid and TargetScan. It showed a complementary match between miR-93 seed sequence and the 3′UTR of TGFβR2 (Figure 2D, Additional file 2: Figure S5). Subsequent dual-luciferase reporter assays reveal that miR-93 significantly attenuated the luciferase activity of reporter vector with the wt 3′UTR of TGFβR2, whereas this effect was abrogated when the 3′UTR-binding site was mutated, supporting that this miRNAs directly regulated TGFβR2 by binding to its 3′UTR (Figure 2D).

According to the relatively high TGFβR2 expression in CNE1 cells and low in CNE2 cells (Additional file 2: Figure S2) as well as relatively low miR-93 expression in CNE1 cells and high in CNE2 cells (Additional file 2: Figure S4), we transfected CNE1 and CNE2 cells with miR-93 mimic and inhibitor, respectively. Both TGFβR2 mRNA and protein expression levels were observed to decline in CNE1 cells and increase in CNE2 cells (Figure 2E, F) accordingly. These data further support that miR-93 directly suppresses TGFβR2 in NPC.

### MiR-93-mediated TGFβR2 down-regulation enhances NPC aggressiveness

To explore the roles of miR-93-mediated TGFβR2 down-regulation in NPC aggressiveness, CNE1 and CNE2 cells were used in both gain- and loss-of-function analyses.

**Figure 3:**
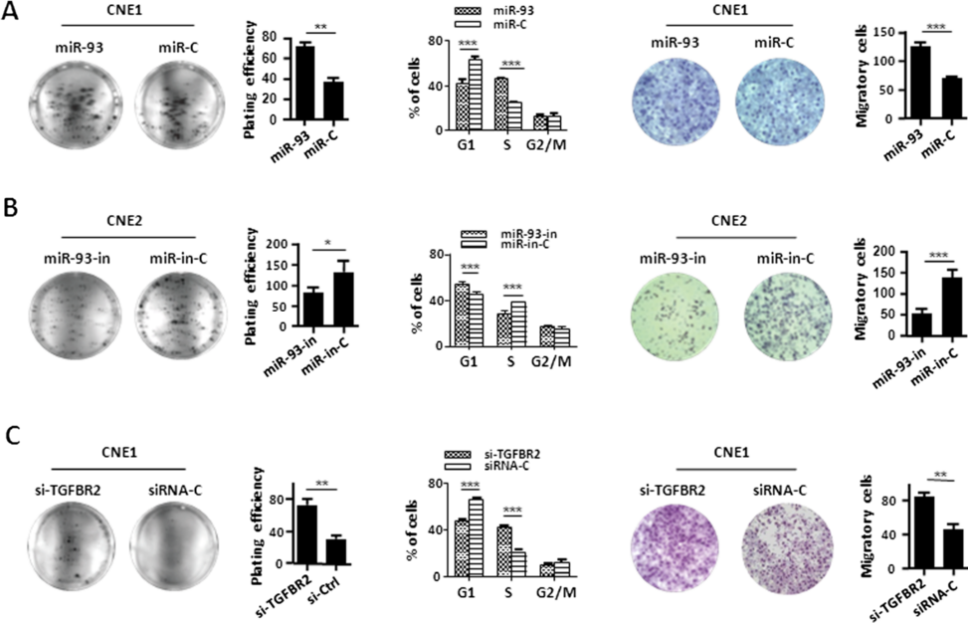
**MiR-93-mediated TGFβR2 down-regulation promotes NPC aggressiveness *in vitro*. (A, C)** Representative results of colony formation, cell cycle distribution, and Transwell migration assays in CNE1 cells treated with miR-93 mimic and siRNA-TGFβR2 respectively. Data are presented as mean ± SEM. (Original magnification 200×). **(B)** Representative results of colony formation, cell cycle distribution, and Transwell migration assays in CNE2 cells treated with miR-93 inhibitor. Data are presented as mean ± SEM. (Original magnification 200×). All results are reproducible in three independent experiments. *P < 0.05, **P < 0.01, ***P < 0.001.

We firstly investigated whether miR-93 was responsible for NPC cell growth and proliferation after regulating TGFβR2 expression (Figure 2E, F). Colony formation assays showed that miR-93 mimic increased cell growth in CNE1 cells, whereas its inhibitor reduced cell growth in CNE2 cells (Figure 3A, B). Consistent results appeared in MTT assays (Additional file 2: Figure S6A, B). Flow cytometry analysis displayed that CNE1 cells transfected with miR-93 mimic exhibited a significantly reduced cell proportion in G1 phase and an increased cell proportion in S-phase. CNE2 cells transfected with miR-93 inhibitor showed opposite alterations (Figure 3A, B, Additional file 2: Figure S7).

We next studied whether miR-93 was also involved in NPC cell invasion and migration after regulating TGFβR2 expression (Figure 2E, F). Cell migration/invasion assays displayed that miR-93 mimic enhanced cell migration and invasion in CNE1 cells (Figure 3A, Additional file 2: Figure S8A), whereas miR-93 inhibitor restricted cell migration and invasion (Figure 3B, Additional file 2: Figure S8B) in CNE2 cells.

Impressively, further investigation showed that the knockdown of TGFβR2 by siRNA (Additional file 1: Table S4, Additional file 2: Figure S10A) enabled a reduced TGFβR2 protein expression (Additional file 2: Figure S10B) along with enhanced proliferation and migration in CNE1 cells (Figure 3C). This phenocopied the effect of miR-93 and supported the major involvement of TGFβR2.

**Figure 4:**
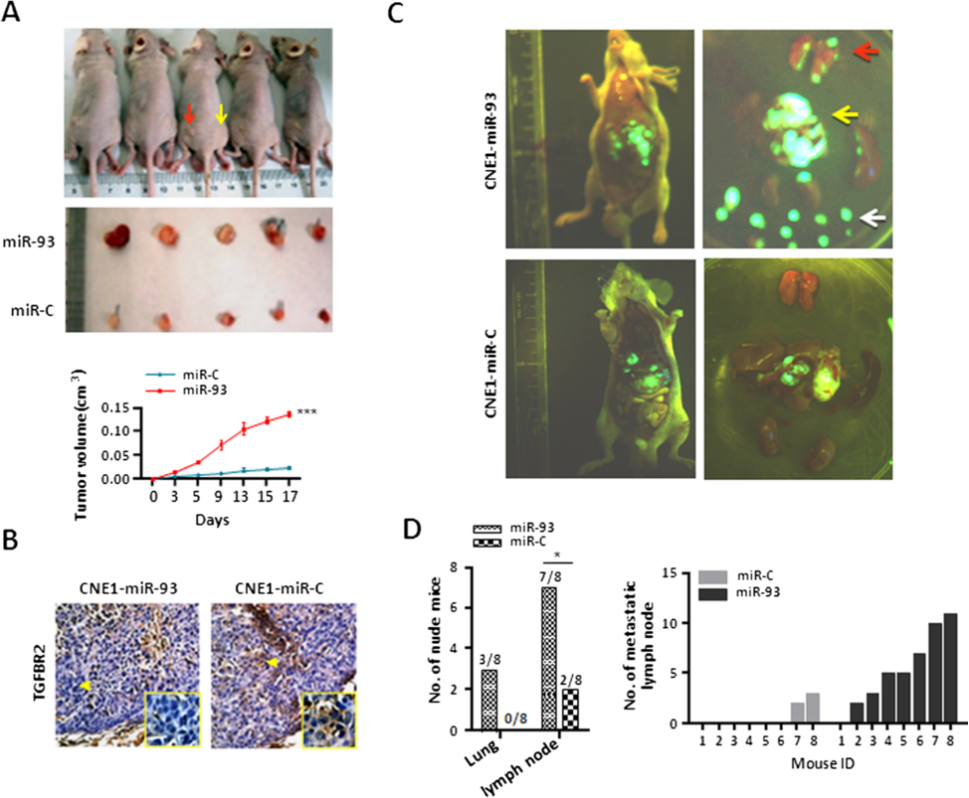
**MiR-93-mediated TGFβR2 down-regulation facilitates NPC aggressiveness *in vivo*. (A)** Tumor cells were subcutaneously injected into nude mice (5 mice each group). Pictures of subcutaneous tumor tissues were showed in whole nude mice injected with CNE1-miR-93 cells (red arrow) and CNE1-miR-C cells (yellow arrow). Tumor volumes were determined by measuring the major (a) and minor (b) diameters at the indicated time points, calculated according to the formula = 0.5 × a × b^2^, and plotted as the mean ± SD. ***P < 0.001. **(B)** TGFβR2 protein expression was examined using IHC in tumor tissues derived from the indicated mouse group (400×). **(C)** The representative GFP images of mouse models transplanted by CNE1-miR-93 cells (upper) and CNE1-miR-C cells (lower). The whole mouse body and resected organs were showed separately. The primary and metastatic tumors were displayed with green fluorescence under whole body imaging system. In the GFP images of resected organs, the lungs (red arrow), liver (yellow arrow), kidneys, and lymph nodes (white arrow) were placed from the upper to bottom respectively and the spleen is at the right side. **(D)** The differences of pulmonary or lymphatic metastasis between two groups (n = 8 per group; Fisher’s exact test. *P < 0.05) and the number of lymph node metastasis of mice (mean ± SD, 5.375 ± 1.349 and 0.625 ± 0.4799 in miR-93 cells and control cells respectively, P = 0.0046).

To support *in vitro* results, we also conducted *in vivo* experiments. The CNE1 cell line stably-expressing miR-93 (CNE1-miR-93) was firstly generated using EGFP-Lenti- miR-93-vector (Additional file 2: Figure S11). Subsequently, we established xenograft mouse models subcutaneously injected with CNE1-miR-93 cells and miR-control cells. We observed that CNE1-miR-93 cells produced bigger tumor volumes in mouse models (Figure 4A). Immunohistochemistry (IHC) on tissue sections derived from tumors showed that TGFβR2 protein expression was reduced in the tumor induced by CNE1-miR-93 cells (Figure 4B). Additionally, we set up NPC metastasis mouse models by transplanting CNE1-miR-93 cells and miR-control cells under liver capsule of mice respectively. The whole body fluorescent imaging system displayed a high metastasis status in a representative CNE1-miR-93 cell-injected mouse. The pulmonary or lymphatic metastasis incidence was obviously higher in CNE1-miR-93 cell-injected mice than in control mice (Figure 4C, D). Notably, miR-93 cells resulted in significantly more lymph node metastases (5.375 ± 1.349) than that of control cells in mouse models (0.625 ± 0.4799) (P = 0.0046) (Figure 4D).

Collectively, these *in vitro* and *in vivo* data suggest that miR-93-mediated TGFβR2 down-regulation promote NPC aggressiveness through enhancing NPC cell proliferation, invasion and metastasis.

### MiR-93 regulates a Smad-dependent and a Smad-independent TGF-β signaling by suppressing TGFβR2

**Figure 5:**
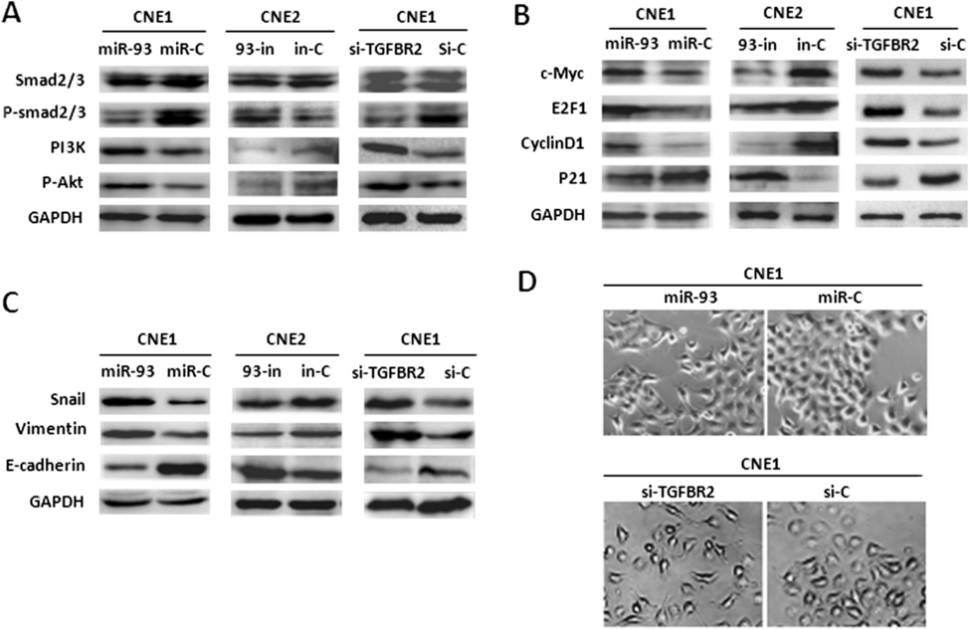
**Regulation of Smad-dependent TGF-β signaling, PI3K/Akt signaling, cell cycle progression and EMT by miR-93-mediated TGFβR2 inactivation in NPC cells. (A)** Western blotting analyses of Smad2/3 and its phosphorylation as well as PI3K and p-Akt in the indicated cell groups. **(B)** Western blotting analyses of C-myc, E2F1, cyclin D1, CDK4 and p21 in the indicated cell groups. **(C)** Western blotting analyses of Snail, E-cadherin, and Vimentin in the indicated cell groups. **(D)** The morphological observations of the indicated cell lines. CNE1 cells were transfected with miR-93 mimic/siRNA-TGFβR2 and miRNA/siRNA control for 96 hours and analyzed under phase contrast microscopy (400×).

TGFβR2 is an important component in TGF-β signaling. Its loss or reduced expression may impair TGF-β signaling. To examine if miR-93 is involved in the abrogation of this signaling pathway, the phosphorylation of Smad2/3, a key protein in this pathway, was detected in CNE1 cells and CNE2 cells treated with miR-93 mimic and inhibitor respectively. As shown in Figure 5A and Additional file 2: Figure S12A, the introduction of miR-93 did attenuate p-Smad2/3 expression, whereas silencing of miR-93 increased p-Smad2/3 expression. Notably, the reduction of p-Smad2/3 expression was also observed in CNE1 treated with siRNA-TGFβR2, similar to the effect of miR-93 (Figure 5A). These data indicate that miR-93 attenuates Smad-dependent TGF-β signal though suppressing TGFβR2.

PI3K/Akt signaling pathway is a central regulator in cancer cell proliferation, metastasis and EMT process. Multiple studies have suggested the existence of direct [52,53] or indirect [54-57] crosstalk between TGF-β signaling and PI3K/Akt signaling, so we wonder whether PI3K/Akt pathway is also involved in NPC aggressiveness in the presence of miR-93-mediated TGFβR2 inactivation. To test this, we examined the expressions of PI3K and p-Akt, after treating CNE1 and CNE2 cells with miR-93 mimic or siRNA-TGFβR2 and miR-93 inhibitor respectively. Our results showed that miR-93 mimic could elevate the expressions of PI3K and p-Akt in CNE1 cells, whereas its inhibitor could reduce the expressions of PI3K and p-Akt in CNE2. As expected, siRNA-TGFβR2 displayed a consentaneous phenocopy with the effect of miR-93 mimic in CNE1 cells (Figure 5A, Additional file 2: Figure S12A), suggesting the down-regulated TGFβR2 could enhance PI3K/Akt pathway.

Next, we performed western blotting to check the expression alterations of some important proteins related to cell proliferation, cell cycle and EMT process in CNE1 cells transfected with miR-93 mimic or siRNA-TGFβR2 and in CNE2 cells transfected with miR-93 inhibitor. Notably, miR-93 mimic or siRNA-TGFβR2 could increase the expression of c-myc, E2F1 and cyclin D1, and reduce p21 expression in CNE1 cells. Conversely, miR-93 inhibitor could reduce the expression of c-myc, E2F1 and cyclin D1, and increase p21 expression in CNE2 cells (Figure 5B, Additional file 2: Figure S12B).

EMT has been regarded as an important mechanism that facilitates cancer cell migration and leads to metastasis, so we also tested whether miR-93 is involved in the EMT to influence cancer metastasis. As shown in Figure 5C and Additional file 2: Figure S12C, both miR-93 mimic and siRNA-TGFβR2 resulted in the highly expressed transcriptional factor Snail, reduced E-cadherin expression and increased Vimentin expression in CNE1 cells, whereas miR-93 inhibitor caused opposite alterations in CNE2 cells. Morphological observation showed that miR-93 mimic or siRNA-treated NPC cells lost their cell-to-cell adhesions and acquired spindle-like morphology, whereas the control cells remained cobblestone-like epithelial appearance (Figure 5D).

Collectively, these data suggest that miR-93-mediated TGFβR2 down-regulation could result in the attenuation of Smad-dependent TGF-β signaling and the activation of PI3K/Akt pathway in NPC aggressiveness. Some cell cycle, cell proliferation and EMT-associated genes were altered in NPC cells in the presence of miR-93-mediated TGFβR2 down-regulation.

TGF-β1 is one of the most important TGF-β signaling components, so we also detected its expression alteration in the presence of miR-93-mediated TGFβR2 down-regulation. Notably, miR-93 or siRNA-TGFβR2 could give rise to an increased TGF-β1 expression in CNE1 cells and an increased TGF-β1 secretion in the culture supernatants of CNE1 cells, whereas miR-93 inhibitor enabled inverse alterations in CNE2 cells (Additional file 2: Figure S9), suggesting a possible feedback loop from miR-93-mediated TGFβR2 to TGF-β1 existed in NPC cells.

### MiR-93 and TGFβR2 were clinically associated with NPC aggressiveness

**Figure 6:**
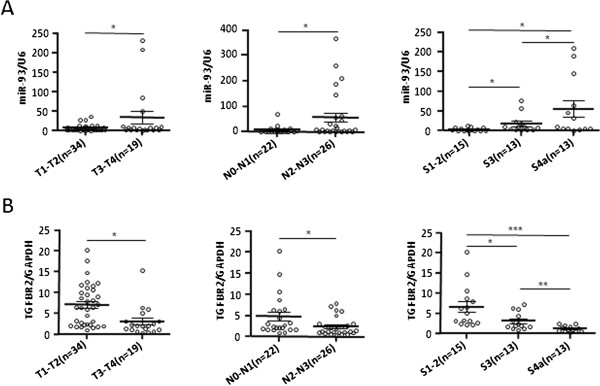
**MiR-93 and TGFβR2 were clinically associated with NPC aggressiveness. (A)** The detection of miR-93 expression by qRT-PCR in an additional set of primary NPC samples. As showed in the plot diagrams, the miR-93 expression was positively correlated with T, N classification and clinical staging. Values represent mean ± SD, *p < 0.05 **(B)** qRT-PCR analysis of TGFβR2 mRNA expression showed a negative correlation of TGFβR2 expression with T and N classifications and clinical staging in scatter plot diagrams. Values represent mean ± SD, *P < 0.05, **P < 0.01, ***P < 0.001.

To further support our finding, we finally investigated the clinical relevance of miR-93 and TGFβR2 in an additional set of clinical samples. The correlations of clinical TNM classification with the expression levels of miR-93 and TGFβR2 were analyzed (M classification was not analyzed due to few patients with distant metastasis). We observed that the expressions of miR-93 positively correlated with T/N classification and clinical stage respectively (Figure 6A) and TGFβR2 expression was negatively correlated with T/N classification and clinical stage respectively (Figure 6B), supporting that miR-93*-*mediated TGFβR2 down-regulation was closely linked to NPC aggressiveness.

## Discussion

The role of TGFBR2 in oncogenesis has been investigated in several cancer types. Loss of TGFβR2 was reported in nasopharyngeal carcinoma (NPC) in our previous study [40]. The downregulation of TGFBR2 expression in cancer cells can be caused by multiple mechanisms, including hypermethylation of the TGFBR2 promoter and, as we show here, through miRNA regulation. MiR-17-92 and its paralogues are the best-known miRNA clusters. Their members have pivotal roles in normal development, and dysregulation of their expressions leads to a wide array of diseases and cancers. In the beginning of our study, we actually failed to find miRNAs targeting TGFBR2 in NPC using a global miRNA expression profiling analysis of clinical samples (data not shown but available in GEO database. Accession Number is GSE42945), similar to other studies [58,59]. Alternatively, based on this miRNA expression profiling data, we next re-classified clinical samples into high and low TGFβR2 expression NPC subgroups and normal control group, and interestingly discovered a cluster set of TGFβR2-associated miRNAs (miR-93, miR-20a, miR-20b and miR-18a), which all belong to miR-17-92 cluster and its paralogues.

To our knowledge, few lines of evidence support that miR-17-92 cluster and its paralogues may contribute to the regulation of TGFβR2 function in cancer. MiR-17-5p and miR-20a could repress TGFβR2 in HCT116 p53-null human colon carcinoma cells [60]*.* Stefano Volinia et al. also experimentally confirmed that TGFBR2 is a target of miR-20a by luciferase reporter assay [61]. Recently, some studies have identified some miR-93 targets that mediate oncogenesis and cancer progression in several different cancer types [30,32,33,35,62]. Our study finds that TGFBR2 is a major miR-93 target that mediates the oncogenic function in NPC.

In the current study, we conducted an integrated approach comprising *in vitro*, *in vivo* and clinical analyses to explore the roles of this miR-93-mediated TGFβR2 down-regulation. Multiple sets of clinical data were used to raise our hypotheses and support our research conclusions. Our results demonstrate that miR-93-mediated TGFβR2 down-regulation contributes to NPC aggressiveness including cell growth and proliferation as well as cancer metastasis and invasion. A recent prognostic study reported a correlation of miR-93 expression level with NPC prognosis [63]. This study didn’t further investigate the associated molecular mechanism, but it supported our findings. Combining this research data with ours, we conclude that up-regulated miR-93 and down-regulated TGFβR2 may be a dominant functional combination in clinical NPC aggressiveness; miR-93 promotes NPC aggressiveness through down-regulating TGFβR2.

It is best known that TGF-β and PI3K/AKT signal transductions are two pivotal pathways that control cell function. Importantly, PI3K/Akt pathway could be directly or indirectly regulated by TGF-β [52-57,64]. In the present study, we provided further evidence that miR-93-mediated TGFβR2 inactivation not only pulled down the TGF-β signaling but also activated PI3K/Akt pathway in NPC aggressiveness. Subsequently, we observed the high expression of two important transcriptional factors, c-myc and snail that are closely associated with cancer cell proliferation, migration and EMT process. The obvious expression alterations of some important genes related to cell proliferation, cell cycle and EMT concurrently appeared in NPC cells in the presence of miR-93-mediated TGFβR2 inactivation. In Particular, the knockdown of TGFβR2 by siRNA displays a consentaneous phenocopy with the effect of miR-93. These date support a major involvement of miR-93-medited TGFβR2 inactivation that drives down the TGF-β signaling and activates PI3K/Akt pathway in NPC aggressiveness.

In addition, a relatively high intracellular expression and secretion of TGF-β1 were observed upon miR-93-mediated TGFβR2 down-regulation, which was consistent with a report by Munoz [65] who believed that it was possibly due to the attenuated TGF-β signaling pathway. This hints its undefined effects on NPC aggressiveness, which deserves further investigations.

## Conclusions

Our study identifies a miR-93-mediated TGFβR2 down-regulation in NPC and its link to NPC aggressiveness. We also discovered an involvement of Smad-dependent TGF-β signaling attenuation and PI3K/Akt activation. This study presents some useful insights into molecular mechanisms underlying NPC aggressiveness and provides potential implications for clinical application.

## Materials and methods

### Clinical samples and cell lines

14 fresh NPC specimens and 8 fresh NP were used for miRNA expression profiling analysis, microarray validation, and correlation analysis. A second cohort of NPC containing 55 fresh-frozen NPC specimens with TNM classification was used for clinical evaluations. 300 paraffin embedded sections from NPC, atypical hyperplasia (AH), normal squamous epithelium (NS) and normal nasopharynx epithelium (NN) were employed for histological examinations. All tissue samples were collected from the patients that were confirmed by pathological examination and not pretreated with radiotherapy or chemotherapy in Zhongshan Hospital, Zhongshan City, Guangdong, China. Staging was performed according to the 1992 Fuzhou NPC staging system of China [66]. Clinical tissue studies for research purposes have received patient’s informed consents and the approval from the Ethics Committee of Southern Medical University, China. Immortalized primary nasopharyngeal epithelial cell line (NP69) and 4 NPC cell lines (CNE1, CNE2, 5-8 F, 6-10B) were obtained from Cancer research institute of Southern medical university, Guangzhou, China.

### MiRNA expression profiling analysis

The miRNA microarrays (CCDTM-miRNA 850-V4p1.4) were provided by Infectious Disease and Immunogenetics Section, DTM, Clinical Center of the National Institutes of Health, USA. Briefly, 7-10 μg of total RNA from each sample was used for hybridization on miRNA microarray that was labeled with Exiqon LNA microRNA Array power labeling kit (Exiqon) according to manufacturer's instruction. The microarray was hybridized at 25°C for 18 hrs, washed in washing buffers at 37°C for 1 min, and scanned on a LuxScan 10 k microarray scanner (LuxScanTM10K-A, CapitalBio Corporation). Resulting data were quantified, normalized and analyzed by the LuxScanTM 3.0 software (CapitalBio Corporation, China), and then uploaded to the mAdb database (http://nciarray.nci.nih.gov).

The raw data were filtered according to standard procedure to exclude spots with minimum intensity and size, normalized using Lowess Smoother, and retrieved by the BRBArray-Tool (http://linus.nci.nih.gov/BRB-ArrayTools.html). Clustering and visualization of expression profiles were preformed with Cluster and Treeview [67].

### Extraction of total RNA and quantitative RT-PCR

Total RNAs were extracted from tissues and cell lines with TRIzol (Invitrogen) according to the user manual. For mRNA expression analysis, 1 μg of total RNA was used for RT using PrimerScriptTM RT Kit following the manufacturers protocol (TaKaRa), and real-time PCR was performed using SYBR® Premix Ex Taq™ Real Time PCR Kit (TaKaRa) on an Mx3000P Stratagene. All data were normalized to GAPDH expression and further normalized to the negative control unless otherwise indicated. Primer sequences for TGFβR2 (forward: 5′-AAGATGACCGCTCTGACATCA-3′, reverse: 5′-CTTATAGACCTCAGCGAC-3′) and GAPDH (forward: 5′-CCATGAGAAGTATGACAAC AGCC-3′, reverse: 5′-GGGTGCTAAGCAG TTGGTG-3′) were acquired from Primer-bank (http://pga.mgh.harvard.edu/primerbank/). For miRNA expression analysis, mature miRNAs were reverse-transcribed for subsequent real-time PCR using All-in-One™ miRNA qRT-PCR Detection Kit following the manufacturer’s protocol (GeneCopoeia). All data were normalized to U6 expression. The fold changes were calculated by relative quantification (2^-∆∆Ct^). QRT-PCR was conducted for each sample in triplicate.

### Cell culture and transfections

NPC cell lines were cultured in RPMI-1640 (HyClone) with 10% calf serum (Gibco). For transfection, the medium was changed to the Dulbecco’s modified Eagle’s medium, DMEM (Gibco) with 10% fetal bovine serum (Gibco). Cells were maintained in a humidified atmosphere of 5% CO_2_ at 37°C and seeded on six-well plates (NEST, China) 24 hours prior to transfection. SiRNA-*TGFβR2*-1515 (Additional file 1: Table S4, Additional file 2: Figure S10) (GenePharma), miRNA-93 mimic and inhibitor (GenePharma) were transfected into cells respectively at a final concentration of 50 nmol/L using Lipofectamine™ 2000 (Invitrogen) in serum-free conditions. After 5 hours, the medium was changed to fresh DMEM (Gibco) with 10% fetal bovine serum.

### Dual luciferase assay

To generate the luciferase reporter constructs psiCHECK2- TGFβR2-3′UTR(wt) and psiCHECK2-TGFβR2-3′UTR(mut), complimentary oligonucleotides containing the wild type 3′UTR of TGFβR2 (3′UTR of TGFβR2 was divided into 2 segments (0.15 KB-0.6 KB and 1.5 KB-2.5 KB) and TGFβR2 3′UTR with point mutations in miR-93 target sites, respectively, were annealed. Oligonucleotide sequences were as follows: TGFβR2-wt 0.15 kb-0.6 kb: forward5′-ATCGCTCGAGCAGCAGGGAGTGGGTGACAT-3′, reverse: 5′-AT CGCGGCCGCTGGCTGTGAGACATGGAGCC-3′. 1.5 kb-2.5 kb: forward: 5′-ATCGTCGAGTC AGTGTGGGTGGGCTGAGA-3′; reverse: 5′-ATCGCGGCCGCGGGAACAGGAGGCAGGATG C-3′. TGFβR2-mut: forward: 5′-AACCGAGGTTCCCGCTCCAAGAAGC-3′, reverse: 5′-CTCCAATGCAGAGGGTAAAA CTATT-3′. Annealed oligonucleotides were ligated into the XhoI/NotI site of psi-CHECK2 renilla/firefly dual-luciferase expression vector. Mutant reporter plasmids were obtained from this plasmid using a KOD-Plus-Mutagenesis Kit (SMK-101, Toyobo Co., Ltd. Life Science Department, Osaka Japan). All inserts were verified by DNA sequencing. Luciferase assays were conducted using 293 T cells plated in a 24-well plate (NEST). Transfections were performed using Lipofectamine™ 2000 (Invitrogen) in OptiMEM serum free media (Gibco).

### Cell migration and invasion assays

For migration assays, 2 × 10^5^ NPC cells transfected with miRNA-93 mimic or siRNA- TGFβR2 and miRNA-93 inhibitor respectively for 48 hours were re-suspended in serum-free media and placed in inserts containing 8 μm pores (Corning) without extracellular matrix coating (BD Biosciences, San Jose, CA, US). DMEM containing 10% FBS was added to the bottom chamber. After 18 hours and 24 hours of incubation, the cells on the lower surface of the filter were fixed and stained followed by microscopic examination. The number of cells in five random optical fields (100× magnifications) from triplicate filters was averaged. For *in vitro* invasion assays, the inserts of the chambers to which the cells were seeded were coated with Matrigel (BD Biosciences).

### Cell proliferation, colony formation and cell cycle analyses

Cell proliferation was analyzed using MTT assay (Sigma, St. Louis, USA). Briefly, CNE-1 cells (5 × 10^3^) and CNE-2 cells (3 × 10^3^) were plated onto 96-well plates (NEST) respectively in 100 μL of growth medium and allowed to adhere overnight. The cells were then transfected with 50 nm of miR-93 mimic or siRNA and inhibitor respectively. At different time points (24 h, 48 h and 72 h), the culture medium was removed and replaced with culture medium containing 10 μL of sterile MTT dye (5 mg/mL). After incubation at 37°C for 4 hours, the MTT solution was removed, and 150 μL of dimethyl sulfoxide (DMSO) was added to dissolve the formazan crystals. Spectrometric absorbance at 490 nm was measured by BioTek ELx800 microplate photometer (BioTek ELx800, SN211805, US).

Colony Formation Assays were performed. CNE-1 and CNE2 cells were transfected respectively with miR-93 mimic or SiRNA-TGFβR2 and miR-93 inhibitor and for 24 h and were pated in 6-well plates at 2×10^2^ for 2 weeks. The plates were then washed twice with PBS, fixed with methanol-acetic acid (3:1 V/V), and stained with 0.5% cristal violet. The number of colonies was counted under the microscope.

For cell cycle analysis, CNE1 cells transfected with miR-93 mimic or siRNA-TGFβR2 and CNE2 cells transfected with miR-93 inhibitor were fixed in 70% ice-cold ethanol for 48 hours at 4°C, stained by incubation with PBS containing 10 μg/mL propidium iodide and 0.5 mg/mL RNase A for 15 min at 37°C, and analyzed for the DNA content of labeled cells by FACS Caliber Cytometry (BD Bioscience). Each experiment was done in triplicate.

### Lentiviral production and transduction

Lentivirus (GV209, H1-MCS-CMV-EGFP) particles carrying hsa-pri-miR-93 precursor and its control were purchased from GeneChem, Shanghai, China [68] (Additional file 2: Figure S11A, B). The lentiviral transduction of CNE1 cells was carried out according to the manufactures’ protocol. The resulting cells were seeded onto 96-well plates and cultured for 3 weeks to produce a stable miR-93-overexpressing CNE1 cells and CNE1 control cells (Additional file 2: Figure S11C). The high expression of miR-93 was validated by quantitative RT-PCR (Additional file 2: Figure S11D).

### Tumor xenografts in nude mice

The experimental protocol was approved by the Animal Care and Use Committee of Southern Medical University. All mice of 4-5 weeks old and 18-20 g in weight were provided by the Central Animal Facility of Southern Medical University.

To evaluate tumor growth in mouse models, 200 μL of Cell suspension from 1 × 10^7^ CNE1 expressing GFP/miR-93 and CNE1 cells expressing vector control were subcutaneously injected into the left and right sides of the back of each mouse respectively. The tumor sizes were measured periodically and calculated using the formula = 0.5 × a × b^2^ (a and b were the long and short diameters of the tumors respectively). After mice were sacrificed in 3 weeks, tumors were collected from mice for IHC detection of TGFβR2 expression.

An imageable technique in mouse models has been developed in our laboratory before [69]. To evaluate tumor metastasis in mouse models, we firstly made a small cut on the abdominal region of each mouse, carefully pushed its liver out of abdominal cavity, injected 50 μL of CNE1 cells (5×10^6^) expressing GFP/miR-93 or an equal number of control cells under the liver envelope of each mouse (8 mice for each group), and then softly pushed its liver back after cleaning and lightly pressing the pinhole with alcohol cotton balls for two minutes. All mice were sacrificed in 3 weeks. Their whole bodies and resected internal organs (on culture plates) were subjected to fluorescent image detection under LT-9MACIMSYSPLUS whole body imaging system (Encinitas, CA, USA).

### Immunohistochemical examination

Paraffin-fixed NPC sections and nude mice tumor sections were immunostained for TGFβR2 using the Ultra-Sensitive S-P IHC Kit (Maixin-Bio, Fujian, China) and primary antibody against TGFβR2 (1:100 dilution, Abcam), and then colorated with DAB Kit (Maixin-Bio, China). The immunohistochemically stained tissue sections were reviewed and scored by two pathologists independently, blinded to the clinical parameters. Staining intensity was scored as previously described [70]. The extent of the staining, defined as the percentage of positive staining areas of tumor cells or normal nasopharyngeal epithelial cells in relation to the whole tissue area, was scored on a scale of 0 to 4 as the following: 0, <10%; 1, 10–25%; 2, 26–50%; 3, 50–75%; and 4, >76%. The sum of the staining-intensity and staining-extent scores was used as the final staining score for TGFβR2 (0–7). For statistical analysis, a final staining scores of 0-5 and 6-7 were respectively considered to be low and high TGFβR2 expression.

### Western blot analysis

Cell lysate was prepared using RIPA buffer with protease inhibitors and quantified using the BCA protein assay (BioTek, China). Protein (20 μg) was loaded onto a 10% SDS-PAGE gel that was then transferred onto PVDF membrane and incubated with anti-TGFβR2 (Bioworld Technology, MN.US), anti-TGF-β1 (Cell Signaling Technology), anti-PI3K (Cell Signaling Technology), anti-p-Akt (p-Ser473, Abzoom), anti-c-myc (Santa Cruz), anti-E2F1 (Santa Cruz), anti-CCND1 (Santa Cruz), anti-p21 (Santa Cruz), anti-E-cadherin (Bioworld Technology, MN.US), anti-Vimentin (Bioworld Technology, MN.US), and anti-Snail (Bioworld Technology, MN.US) at 4°C overnight in blocker (3% non-fat dry milk/BSA in TTBS) followed by incubation with HRP-conjugated secondary anti mouse (ZSGB-Bio, China). Protein was normalized with GAPDH (Abmart).

### Enzyme-linked immunosorbent assay (ELISA)

CNE1 cells transfected with miR-93 mimic or siRNA-TGFβR2 and CNE2 cells transfected with miR-93 inhibitor were incubated in 8 ml RPMI-1640 (HyClone) with 10% calf serum (Gibco) for 48 hours. When cells were harvested, the media were placed in the ELISA plates (KeyGen Biotech Co. Ltd, China). ELISA of TGF-β1 was performed following the routine manual.

### Statistical analysis

All statistical analyses were performed by the SPSS 13.0 statistical software package (SPSS Inc. Chicago, IL, USA). The Kruskal Wallis test and χ^2^ test were used to compare gene expression levels of different histological types. The χ^2^ test was used to analyze the relationship between the levels of TGFβR2 expression and clinicopathologic characteristics. Survival curves were plotted using the Kaplan-Meier method and compared using the log-rank test. The significances of various variables in survival were analyzed using the multivariate Cox proportional hazards model. Two-tailed Student’s t test was used to determine the difference between two groups, while ANOVA was used for the comparison of more than two groups. Fisher’s exact test was employed when sample sizes were relatively small. The differences were considered to be statistically significant when p-value <0.05. All data were presented as mean ± SD or SEM unless otherwise noted.

## Competing interests

The authors declare that they have no competing interest.

## Authors’ contributions

XL, JLL, HBC, and WYF designed the experiment, interpreted the data and prepared the manuscript. XML, HZ and LMC conducted the experiment, collected the data and helped to prepare the manuscript. YFY, LZ, JBL, WSC, EW, FM.M, interpreted the data. KTY was a key advisor of this project. All authors read and approved the final manuscript.

## Supplementary Material

Additional file 1: Table S1 The Correlation between clinicopathologic features and TGFβR2 protein expression in NPC. **Table S2.** Summary of univariate and multivariate Cox regression analysis of overall survival duration. **Table S3.** Differentially expressed miRNAs in three subgroups of tissue samples. **Table S4.** The information of TGFBR2 interference fragments. Click here for file

Additional file 2: Figure S1 Kaplan–Meier survival analysis of overall survival correlated to NPC TNM classification. **Figure S2.** QRT-PCR and Western blot analyses of TGFβR2 expression in NPC cell lines and NPC tissue. **Figure S3.** TGFβR2 expression levels in three NPC subgroups. **Figure S4.** miR-93 expression in NPC cell lines and immortalized Nasopharyngeal epithelial cell, NP69. **Figure S5.** TargetScan prediction of miRNAs targeting 3′ UTR of TGFβR2 gene. **Figure S6.** Effect of miR-93 mimic, inhibitor and siRNA-TGFβR2 on cell proliferation as detected by MTT assay. **Figure S7.** Flow cytometry analysis by FACS Caliber cytometry. **Figure S8.** MiR-93-mediated TGFβR2 down-regulation promotes NPC cell invasion. **Figure S9.** miR-93-mediated TGFβR2 down-regulation results in a relatively higher level of TGF-β1 intracellular expression and secretion. **Figure S10.** The interference efficiency of TGFβR2 interference fragments. **Figure S11.** Lentiviral vectors (miR-93\GV209 and miR-ctrl\GV209) were constructed for the transfection. **Figure S12.** The histograms of the quantification for the western bands. Click here for file
