# Supplementary material for: TGFβR2 is a major target of miR-93 in nasopharyngeal carcinoma aggressiveness
Source: Mol Cancer. 2014 Mar 8;13:51. doi: 10.1186/1476-4598-13-51 (PMC4016586; doi:10.1186/1476-4598-13-51)
Supplement: Additional file 1: Table S1 — The Correlation between clinicopathologic features and TGFβR2 protein expression in NPC. Table S2. Summary of univariate and multivariate Cox regression analysis of overall survival duration. Table S3. Differentially expressed miRNAs in three subgroups of tissue samples. Table S4. The information of TGFBR2 interference fragments. [file 1476-4598-13-51-S1.pdf]

Supplementary Table 1 The Correlation between clinicopathologic features and TGFβR2 protein expression in NPC

Characteristics	n	TGFBR2 (%)		*P*
		High expression	Low expression	
Gender				
Male	103	50(48.5)	53 (51.5)	
Female	53	28(52.8)	25 (47.2)	0.735
Age(y)				
≥50	71	31 (43.7)	40 (56.3)	
＜50	85	47 (55.3)	38(44.7)	0.198
Smoking				
Yes	31	18 (58.1)	13 (41.9)	
No	125	60 (48.0)	65 (52.0)	0.423
Family tumor history				
Yes	5	4(80.0)	1(20.0)	
No	151	74(49.0)	77(51.0)	0.367
Recurrence				
Yes	39	17(43.6)	22(56.4)	
No	117	61(52.1)	56(47.9)	0.460
T classification				
T_1-T_2	109	62(56.9)	40 (43.1)	
T_3-T_4	47	16 (51.7)	38(48.3)	<0.001
N classification				
N_0-N_1	87	45 (51.7)	28(48.3)	
N_2-N_3	69	33(47.8)	50 (52.2)	0.010
Distant metastasis [#]				
Yes	9	3 (33.3)	6 (66.7)	
No	147	75 (51.0)	72 (49.0)	0.495
TNM Clinical stage				
I-II	47	31 (66.0)	16 (34.0)	
III-IV	109	47(43.1)	62(56.9)	0.014

[#] The difference that was not statistically significant, possibly due to few patients with distant metastasis

Supplementary Table 2 Summary of univariate and multivariate Cox regression analysis of overall survival duration

Parameter	Univariate analysis			Multivariate analysis		
	P	HR	95%CI	*P*	HR	95%CI
Gender						
Male vs. female	0.880	1.041	0.617-1.755			
Age						
≥50 vs.＜50 years	0.073	1.576	0.959-2.592			
Family tumor history						
Yes vs. No	0.369	0.404	0.056-2.917			
Smoking						
Yes vs. No	0.536	0.814	0.425-1.560			
Biotherapy						
Yes vs. No	0.554	0.653	0.159-2.677			
T classification						
T_1-T_2 vs. T_3-T_4	<0.001	2.574	1.573-4.215	0.040	1.867	1.030-3.383
N classification						
N_0-N1 vs. N_2-N_3	<0.001	3.553	2.011-6.277	0.002	3.562	1.583-8.014
M classification						
M_0 vs. M_1	<0.001	6.367	3.002-13.507	0.000	7.689	3.388-17.448
Clinical stage						
I-II vs. III-IV	<0.001	3.563	1.755-7.234	0.628	0.759	0.249-2.314
TGFBR2 expression						
High vs. low	<0.001	0.181	0.098-0.333	0.000	0.239	0.125-0.458

Supplementary Table 3. Differentially expressed miRNAs in three subgroups of tissue samples

Description	Parametric p-value	FDR	Permutation p-value	Geom mean of ratios			Unique id
				group 1	group 2	Group 2	
hsa-miR-20a	0.00339	0.1932708	0.0012	0.489626	0.4907058	0.9801892	6519885
hsa-miR-93	0.00621	0.1932708	0.0025	0.5247165	0.5789282	1.0329631	6520210
hsa-miR-20b	0.00793	0.1932708	0.0095	0.5672913	0.4738735	1.3353771	6520264
hsa-miR-18a	0.04642	0.3629741	0.0727	0.2738743	0.3217721	0.6608565	6520174

Supplementary Table 4 The information of TGFBR2 interference fragments

The interference fragment	Base sequence
SiRNA-TGFBR2-homo-462	Sense ; 5'-GUUCAGAAGUCGGUUAAUATT-3'<br>Antisense ; 5'-UAUUAACCGACUUCUGAACTT-3'
SiRNA-TGFBR2-homo-663	Sense ; 5'-GUGGCUGUAUGGAGAAAGATT-3'<br>Antisense ; 5'-UCUUUCUCCAUACAGCCACTT-3'
SiRNA-TGFBR2-homo-796	Sense ; 5'-GCUCUGAUGAGUGCAAUGATT-3'<br>Antisense ; 5'-UCAUUGCACUCAUCAGAGCTT-3'
SiRNA-TGFBR2-homo-1141	Sense ; 5'-GCUUUGCUGAGGUCUAUAATT-3'<br>Antisense ; 5'-UUAUAGACCUCAGCAAAGCTT-3'
SiRNA-TGFBR2-homo-1515	Sense ; 5'-GACCUCAAGAGCUCCAAUATT-3'<br>Antisense ; 5'-UAUUGGAGCUCUUGAGGUCTT-3'
SiRNA-negative control	Sense ; 5'-UUCUCCGAACGUGUCACGUTT-3'<br>Antisense ; 5'-ACGUGACACGUUCGGAGAATT-3'
