# Supplementary material for: TGFβR2 is a major target of miR-93 in nasopharyngeal carcinoma aggressiveness
Source: Mol Cancer. 2014 Mar 8;13:51. doi: 10.1186/1476-4598-13-51 (PMC4016586; doi:10.1186/1476-4598-13-51)
Supplement: Additional file 2: Figure S1 — Kaplan–Meier survival analysis of overall survival correlated to NPC TNM classification. Figure S2. QRT-PCR and Western blot analyses of TGFβR2 expression in NPC cell lines and NPC tissue. Figure S3. TGFβR2 expression levels in three NPC subgroups. Figure S4. miR-93 expression in NPC cell lines and immortalized Nasopharyngeal epithelial cell, NP69. Figure S5. TargetScan prediction of miRNAs targeting 3′ UTR of TGFβR2 gene. Figure S6. Effect of miR-93 mimic, inhibitor and siRNA-TGFβR2 on cell proliferation as detected by MTT assay. Figure S7. Flow cytometry analysis by FACS Caliber cytometry. Figure S8. MiR-93-mediated TGFβR2 down-regulation promotes NPC cell invasion. Figure S9. miR-93-mediated TGFβR2 down-regulation results in a relatively higher level of TGF-β1 intracellular expression and secretion. Figure S10. The interference efficiency of TGFβR2 interference fragments. Figure S11. Lentiviral vectors (miR-93\GV209 and miR-ctrl\GV209) were constructed for the transfection. Figure S12. The histograms of the quantification for the western bands. [file 1476-4598-13-51-S2.pdf]

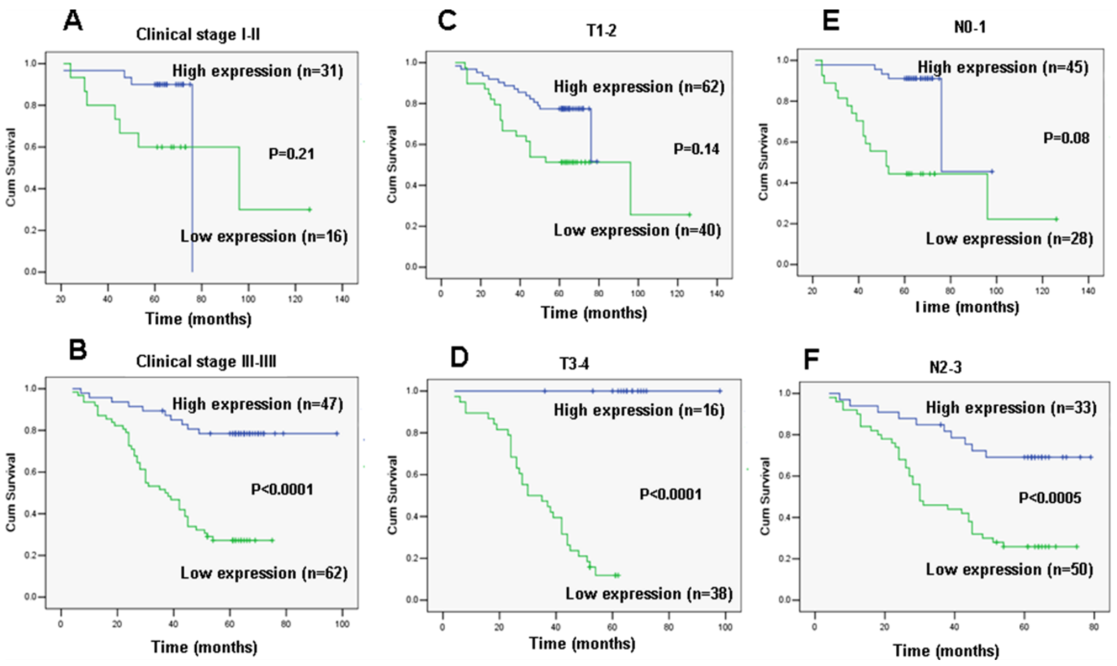

Supplementary Figure 1 Kaplan–Meier survival analysis of overall survival correlated to NPC TNM classification. (**A**, **B**) Cumulative survival in early-stage patients and late-stage patients according to TGFβR2 expression. (**C**, **D**) Survival analysis in T1-2 patients and T3-4 patients according to TGFβR2 expression. (**E**, **F**）Survival analysis in N0-1 patients and N2-3 patients according to TGFβR2 expression. The log-rank test was used to calculate p values.

A

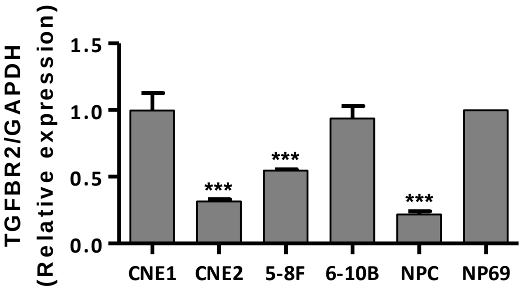

B

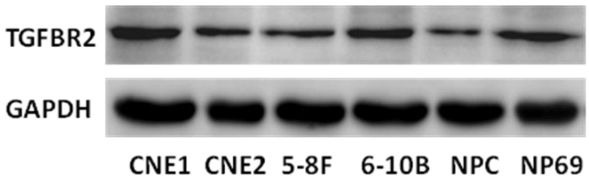

Supplementary Figure 2 (**A**) QRT-PCR analysis of TGFβR2 expression in the indicated NPC cell lines and NPC tissue (3 NPC samples were pooled). Values represent mean ± SD; n = 3. ***P<0.001. (**B**) Western blotting analysis of TGFβR2 expression in the indicated NPC cells, NPC tissue (3 NPC samples were pooled) and NP69 cells, GAPDH was used as a loading control.

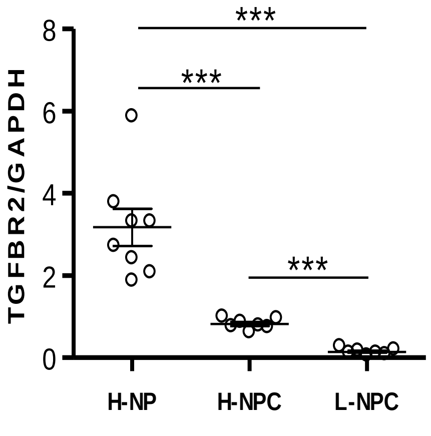

Supplementary Figure 3 TGFβR2 expression levels in three subgroups including 8 NP with high TGFβR2 expression (H-NP), 7 NPC samples with high TGFβR2 expression (H-NPC), and 7 NPC samples with low TGFβR2 expression(L-NPC). The means ± SD were 3.173 ± 0.4511, 0.821 ± 0.05008 and 0.146 ± 0.02816, respectively. *** P < 0.001.

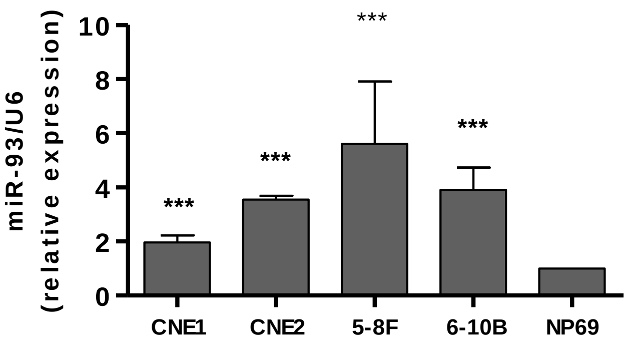

Supplementary Figure 4 miR-93 expression in NPC cell lines (CNE1, CNE2, 5-8F and 6-10B) and immortalized Nasopharyngeal epithelial cell, NP69. Values represent mean ± SD; n = 3. ***P < 0.001.

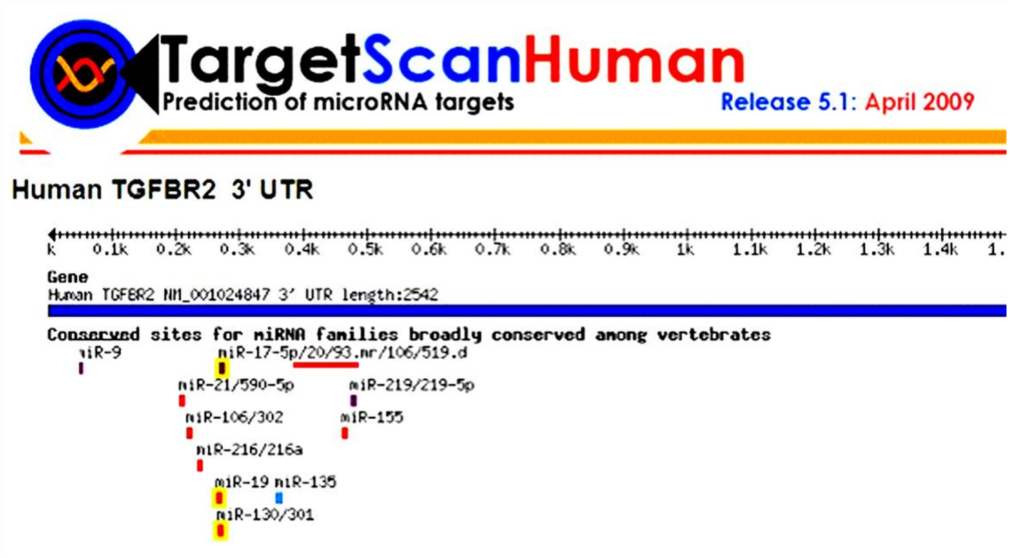

Supplementary Figure 5 TargetScan prediction of miRNAs targeting 3' UTR of TGFβR2 gene. A couple of candidate miRNA binding sites including miR-93 in the 3'UTR of TGFβR2. TargetScan is release 5.1 (http://targetscan.org/) .

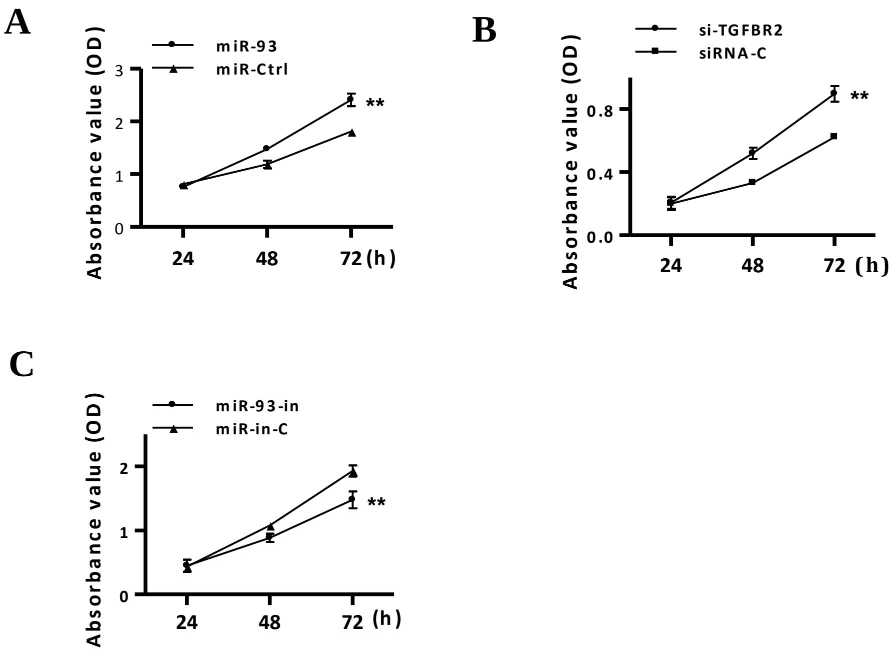

Supplementary Figure 6 Effect of *miR-93* mimic, inhibitor and siRNA-TGFβR2 on cell proliferation as detected by MTT assay in indicated cells. Data are presented as mean ± SEM for three independent experiments. **p <0.01.

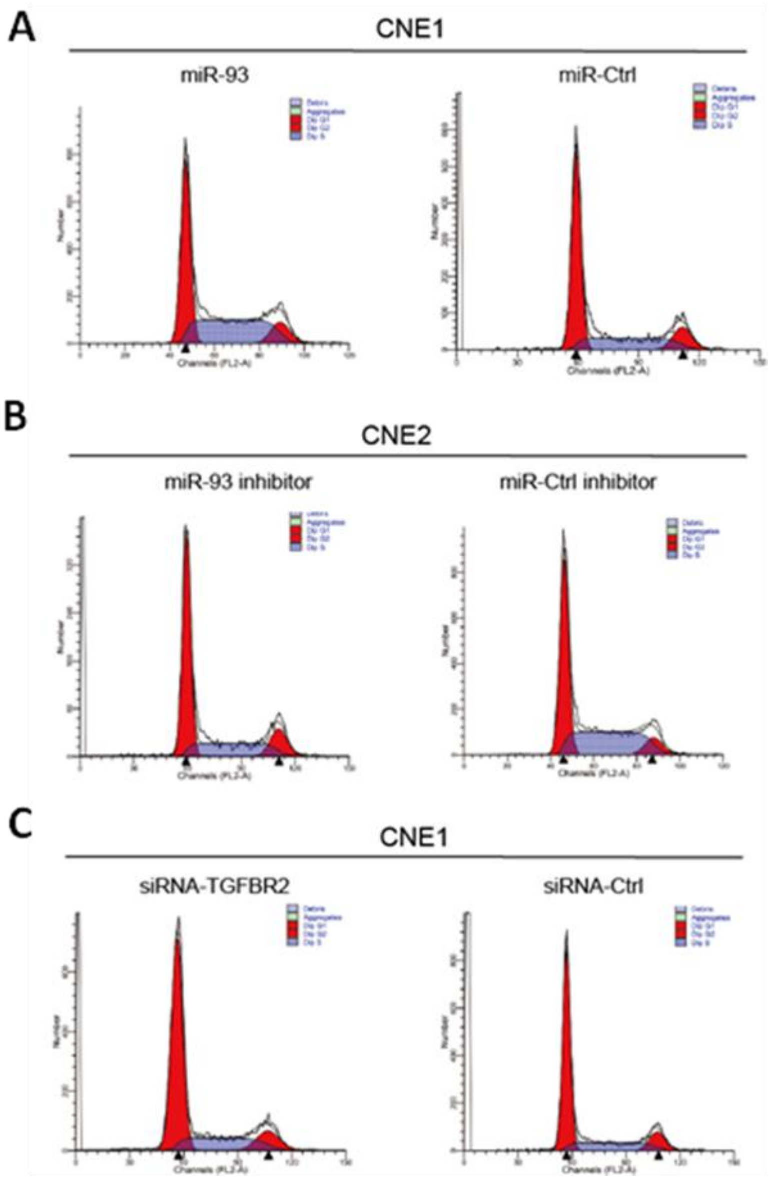

Supplementary Figure 7 Flow cytometry analysis by FACS Caliber cytometry. (**A, C**) The cell cycle distribution in CNE1 cells transfected with miR-93 mimic, siRNA-TGFβR2 and miR-/siRNA-Ctrls. (**B**) The cell cycle distribution in CNE2 cells transfected with miR-93 inhibitor and miR-control.

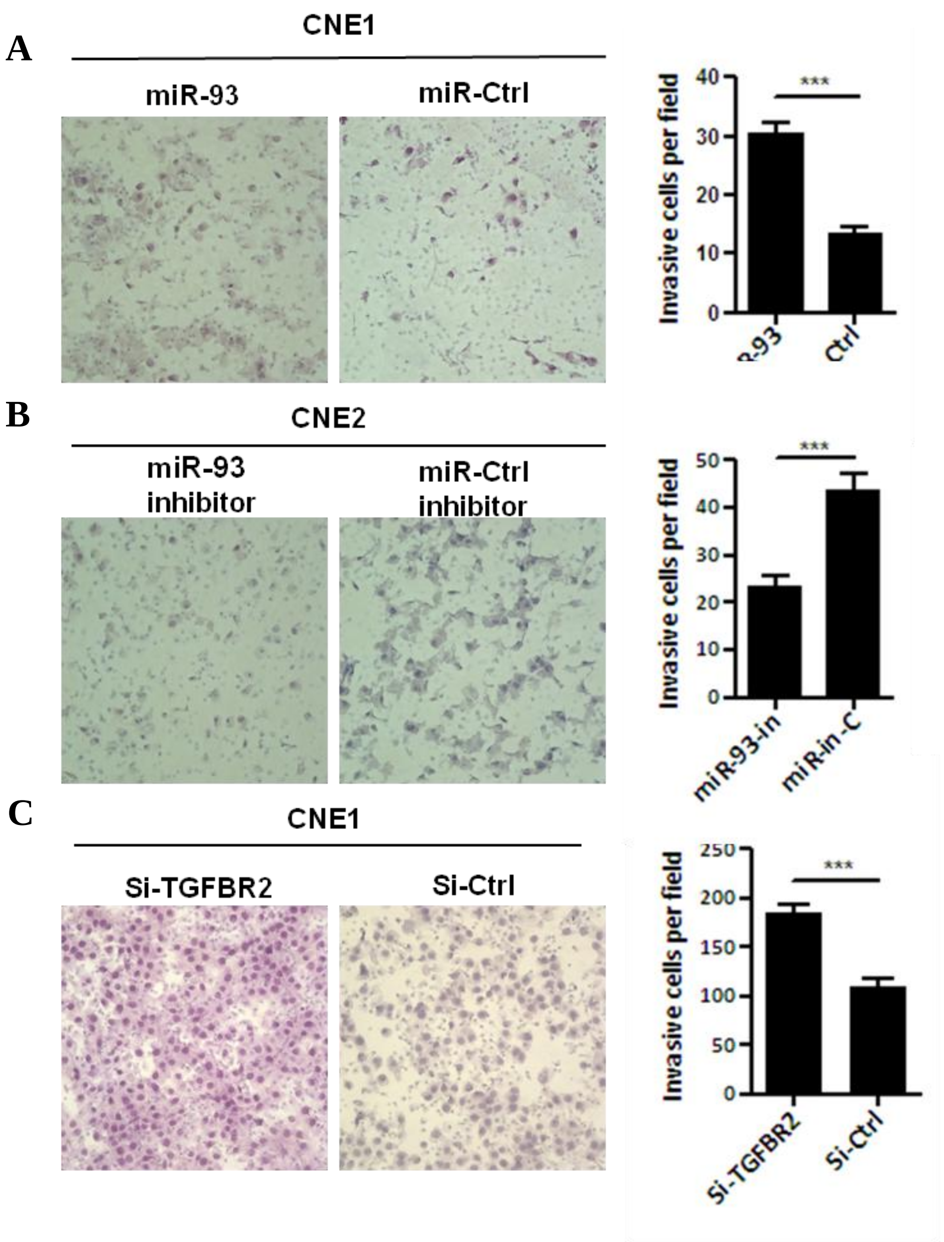

Supplementary Figure 8 MiR-93-mediated TGFβR2 down-regulation promotes NPC cell invasion. (**A**, **C**) The invasion assays (in a modified Boyden chamber) in the indicated CNE1 cells. (**B**) The invasion assays in the indicated CNE2 cells. Data are presented as mean ± SEM of cells attached to the lower surface of the membrane in different cell groups for three independent experiments (400×). *** P<0.001.

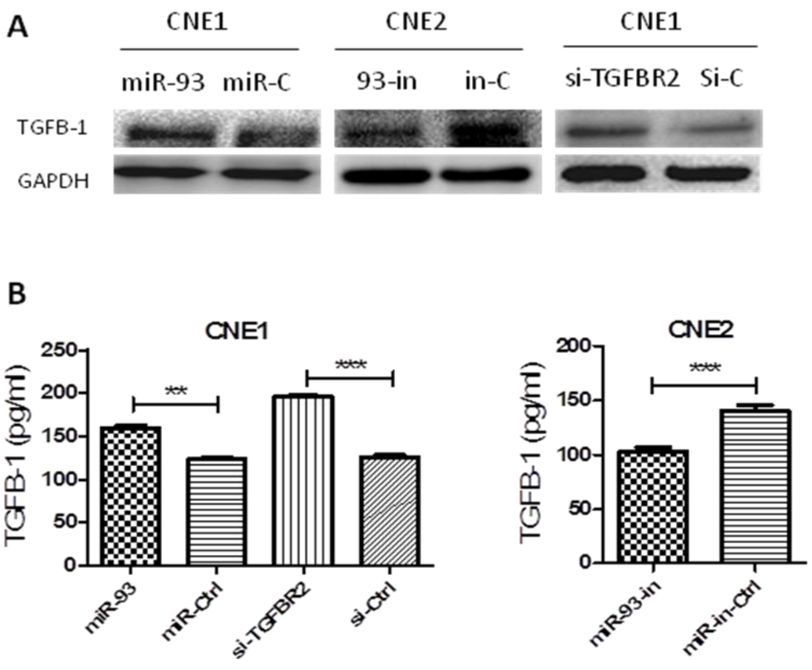

Supplementary Figure 9 miR-93-mediated TGFβR2 down-regulation results in a relatively higher level of TGF-β1 intracellular expression and secretion. **(A)** Western blotting analysis of TGF-β1 expression in the indicated cell groups. GAPDH was used as a loading control. **(B)** ELASA assay of TGF-β1 concentration in the culture supernatants of indicated cells in triplicate. * $P<0.05$, **$P<0.01$, ***$P<0.001$.

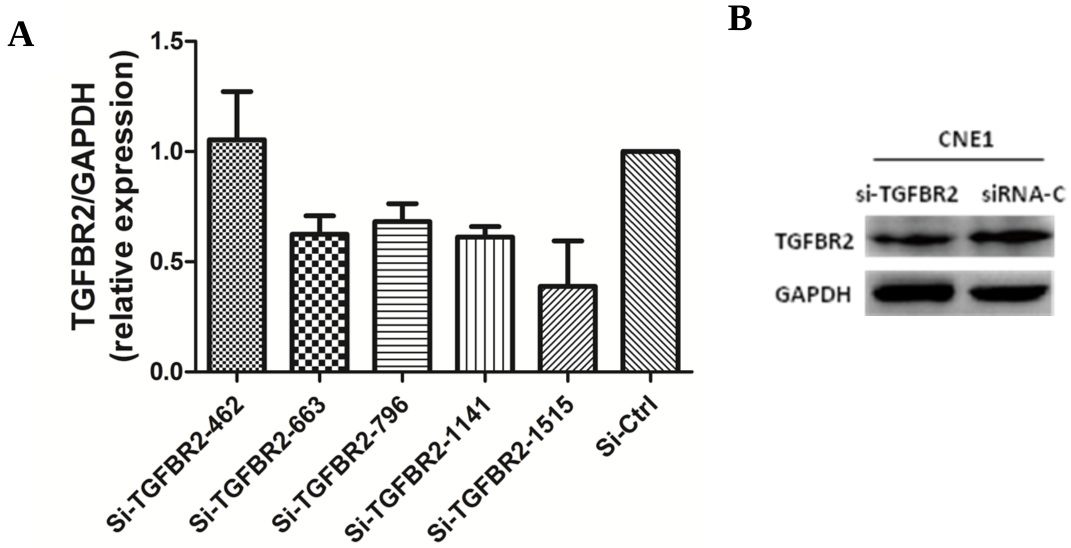

Supplementary Figure 10 The interference efficiency of TGFβR2 interference fragments. **(A)** QRT-PCR analysis of the TGFβR2 expression in the indicated CNE1 cells. Values represent mean ± SD; n = 3. **(B)** Western blotting of TGFβR2 protein expression in CNE1 cells treated with si-TGFβR2-1515 interference fragments.

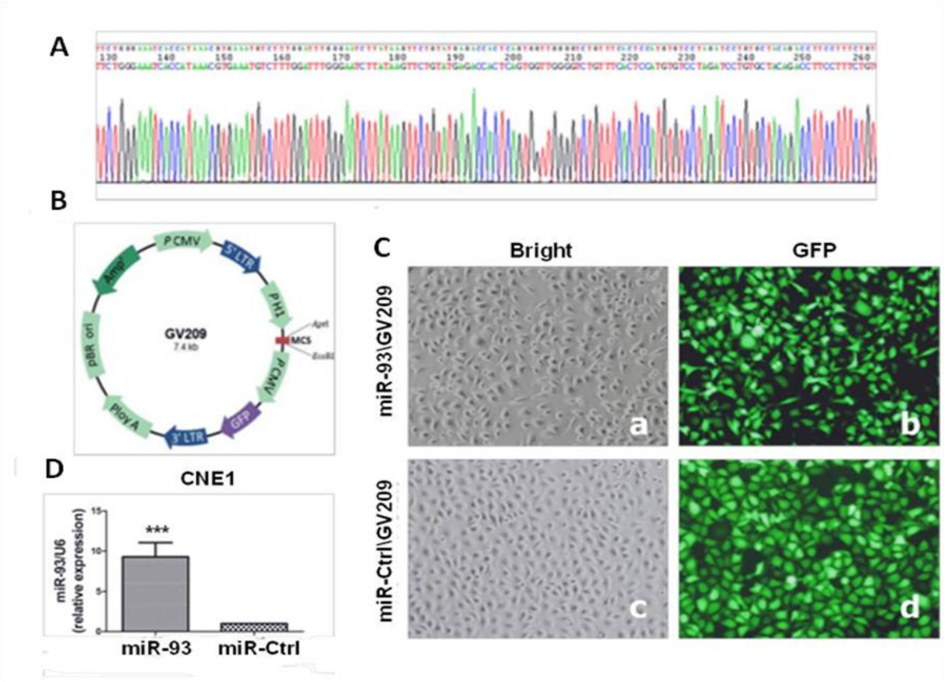

Supplementary Figure 11 Lentiviral vectors (miR-93\GV209 and miR-ctrl\GV209) were constructed for the transfection. (**A**) Sequencing result of miR-93\GV209 derived from the recombinant plasmid. (**B**) Structure of GV209 (H1-MCS-CMV-EGFP). (**C**) CNE1 cells transfected with miR-93\GV209 were observed under (a) visible light and (b) fluorescence microscope (200×) Cells treated by miR-ctrl\GV209 were observed under (c) visible light and (d) fluorescence microscope (200×). (**D**) Detection of miR-93 expression in the indicated cells by qRT-PCR. ***P<0.001.

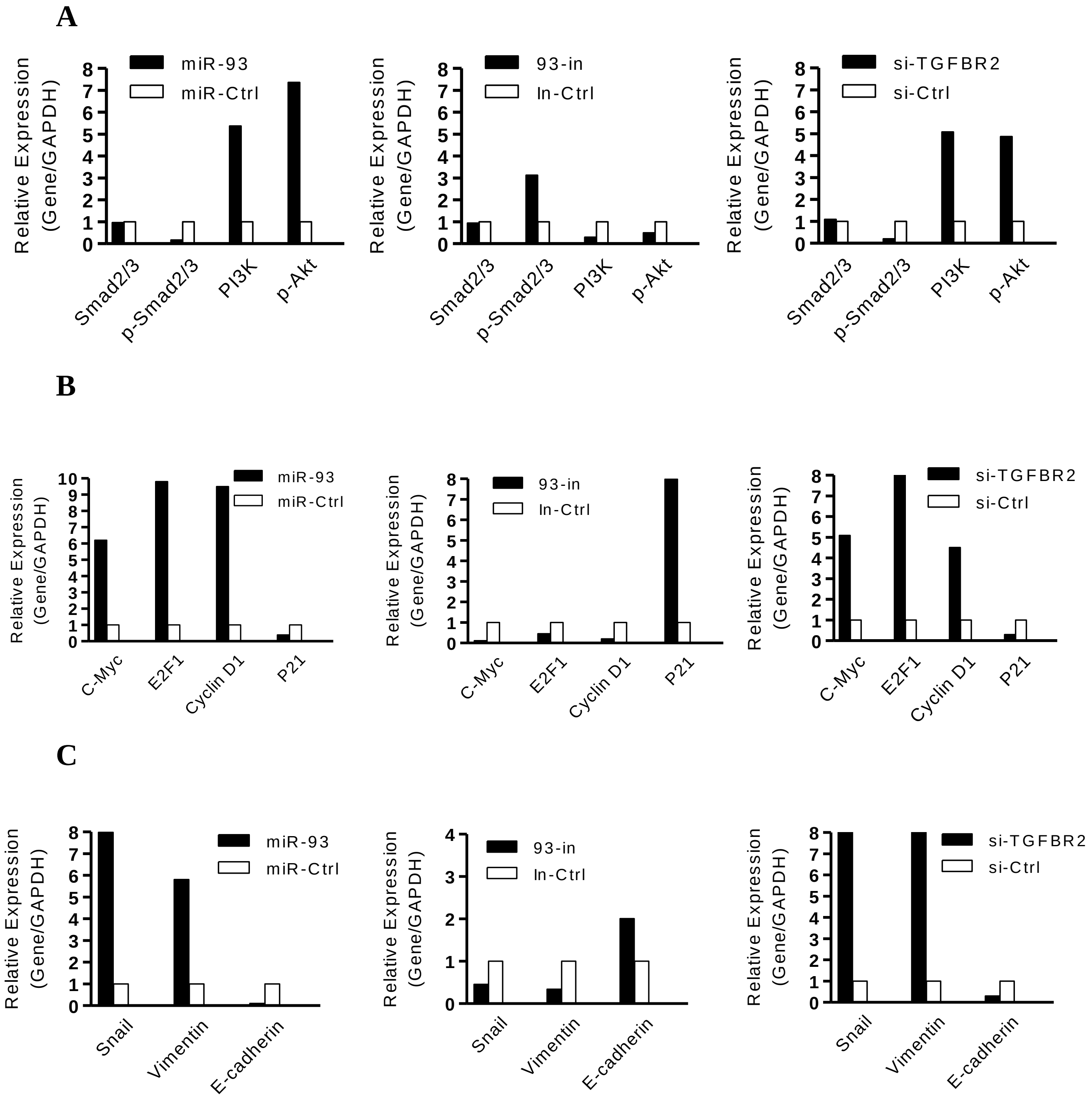

Supplementary Figure 12 The histograms of the quantification for the western bands. (**A**) Western blotting analyses of Smad2/3 and its phosphorylation as well as PI3K and p-Akt in the indicated cell groups. (**B**) Western blotting analyses of C-myc, E2F1, cyclin D1, CDK4 and p21 in the indicated cell groups. (**C**) Western blotting analyses of Snail, E-cadherin, and Vimentin in the indicated cell groups. All of gene expression levels were semi-quantified by analysis of the Western blot with Gel-Pro Analyzer software.
